# Supplementary material for: The Disproportionate Rise in Pancreatic Cancer in Younger Women Is Due to a Rise in Adenocarcinoma and Not Neuroendocrine Tumors: A Nationwide Time-Trend Analysis Using 2001–2018 United States Cancer Statistics Databases
Source: Cancers (Basel). 2024 Feb 28;16(5):971. doi: 10.3390/cancers16050971 (PMC10931165; doi:10.3390/cancers16050971)
Supplement: Supplementary file 1 [file cancers-16-00971-s001.zip › cancers-2869765-supplementary.pdf]

Supplemental table S1: Sex-specific Trends for Biopsy-proven Pancreatic Ductal Adenocarcinoma (PDAC) and Pancreatic Neuroendocrine Tumor (PanNET) Stratified by Age Groups

		Trends					Comparison between sex-specific trends (p-value)[e]			
Age group (years)	Number of cases (%)	Time period	APC (95% CI)[a]	APC p-value	AAPC (95% CI)	AAPC p-value	AAPC difference (95% CI)[b]	AAPC diff p-value	Test of coincidence[c]	Test of parallelism[d]
				Pancreatic Ductal Adenocarcinoma (PDAC)						
**All Ages**	**N = 554,215**									
Men	288,063 (52.0%)	2001-2006	1.94* (1.43-2.45)	<0.001	1.35* (1.20-1.51)	<0.001	-0.17 (-0.39-0.04)	0.12	<0.001	0.004
		2006-2018	1.11* (1.00-1.22)	<0.001						
Women	266,152 (48.0%)	2001-2008	2.17* (1.84-2.49)	<0.001	1.52* (1.38-1.67)	<0.001				
		2008-2018	1.08* (0.91-1.24)	<0.001						
**Age ≥55**	**N= 488,009**									
Men	250,162 (51.3%)	2001-2005	2.25* (1.43-3.07)	<0.001	1.54* (1.35-1.73)	<0.001	-0.024 (-0.27-0.22)	0.85	<0.001	0.015
		2005-2018	1.32* (1.21-1.43)	<0.001						
Women	237,847 (48.7%)	2001-2008	2.24* (1.89-2.58)	<0.001	1.56* (1.41-1.72)	<0.001				
		2008-2018	1.09* (0.93-1.26)	<0.001						
**Age <55**	**N= 66,097**									
Men	37,873 (57.3%)	2001-2018	0.14 (-0.12-0.40)	0.26	0.14 (-0.12-0.40)	0.26	-1.05* (-1.42- -0.67)	<0.001	<0.001	<0.001
Women	30,245 (42.7%)	2001-2018	1.19* (0.88-1.49)	<0.001	1.19* (0.88-1.49)	<0.001				
**Ages 35-54**	**N = 64,066**									
Men	37,056 (57.8%)	2001-2006	1.45 (0.01-2.92)	0.05	0.25 (-0.20-0.70)	0.28	-0.69 (-1.23 - -0.15)	0.01	<0.001	0.002
		2006-2018	-0.25 (-0.61-0.12)	0.17						
Women	27,010 (42.2%)	2001-2018	0.94* (0.63-1.25)	<0.001	0.94* (0.63-1.25)	<0.001				
**Age 15-34**	**N= 2031**									
Men	817 (40.2%)	2001-2018	1.12 (-0.32-2.57)	0.12	1.12 (-0.32-2.57)	0.12	-8.64 (-14.4 - -2.93)	<0.01	<0.001	0.005
Women	1214 (59.8%)	2001-2003	42.9 (-8.78-123.7)	0.11	9.76* (4.35-15.5)	<0.001				
		2003-2013	3.49* (0.54-6.52)	0.03						
		2013-2018	11.1* (5.50-17.0)	0.001						
				Pancreatic Neuroendocrine Tumor (PanNET)						
**All ages**	**N = 41,889**									
Men	22,918 (54.7%)	2001-2010	7.36* (6.35-8.38)	<0.001	7.55* (6.20-8.92)	<0.001	0.20 (-1.79-2.18)	0.85	<0.001	0.65
		2010-2013	16.3* (8.13-25.1)	0.001						
		2013-2018	2.96* (1.58-4.36)	0.001						
Women	18,971 (45.3%)	2001-2010	6.90* (5.85-7.96)	<0.001	7.36* (5.92-8.81)	<0.001				
		2010-2013	15.7* (6.99-25.0)	0.002						
		2013-2018	3.45* (1.96-4.95)	<0.001						
**Age ≥55**	**N= 29,089**									
Men	16,475 (56.6%)	2001-2009	6.97* (6.05-7.90)	<0.001	7.96* (7.19-8.73)	<0.001	0.15 (-2.01-2.31)	0.89	<0.001	0.02
		2009-2013	15.7* (12.5-18.9)	<0.001						
		2013-2018	3.65* (2.70-4.61)	<0.001						
Women	12,614 (43.4%)	2001-2010	7.68* (6.15-9.22)	<0.001	7.80* (5.81-9.84)	<0.001				
		2010-2013	14.5* (2.84-27.6)	0.02						
		2013-2018	4.18* (2.18-6.22)	0.001						
**Age <55**	**N= 12,744**									
Men	6,416 (50.3%)	2001-2016	7.97* (6.94-9.02)	<0.001	6.30* (4.16-8.49)	<0.001	-0.79 (-4.21-2.62)	0.65	0.47	0.58
		2016-2018	-5.45 (-20.8-12.8)	0.51						
Women	6,328 (49.7%)	2001-2003	15.4 (-4.87-40.0)	0.12	7.09* (4.48-9.77)	<0.001				
		2003-2009	3.44 (-0.20 -7.21)	0.06						
		2009-2014	14.2*(9.60 – 18.9)	<0.001						
		2014-2018	0.34 (-3.16-3.97)	0.83						

Age group (years)	Number of cases (%)	Time period	APC (95% CI)	APC p-value	AAPC (95% CI)	AAPC p-value	AAPC difference (95% CI)	AAPC difference	Test of coincidence	Test of parallelism
**Age 35-54**	**N= 11,044**									
Men	5,683 (51.5%)	2001-2016	7.97* (6.94-9.02)	<0.001	6.30* (4.16-8.49)	<0.001	-0.79 (-4.21-2.63)	0.65	0.004	0.15
		2016-2018	-5.45 (-20.8 - -12.8)	0.51						
Women	5,361 (48.5%)	2001-2003	15.4 (-4.87 – 40.0)	0.12	7.09* (4.48-9.77)	<0.001				
		2003-2009	3.44 (-0.20 – 7.21)	0.06						
		2009-2014	14.2* (9.60 - 18.9)	<0.001						
		2014-2018	0.34 (-3.16 – 3.97)	0.83						
**Age 15-34**	**N= 1700**									
Men	733 (43.1%)	2001-2018	7.24* (5.73-8.77)	<0.001	7.24* (5.73-8.77)	<0.001	-0.69 (-2.92-1.55)	0.55	<0.001	0.60
Women	967 (56.9%)	2001-2018	7.92* (6.06-9.81)	<0.001	7.92* (6.06-9.81)	<0.001				

a. APC and AAPC refer to "annual percentage change" and "average annual percentage change," respectively.

* indicates a significant difference (p < 0.05). Trends were calculated using version 4.9 of the Joinpoint Regression program (National Cancer Institute). Up to four joinpoints (5 line segments) were allowed. The annual percentage change over the whole period (2001-2018) is equal to the average over all the subgroups.

b. Negative value indicates greater average APC in women.

c. Tests whether sex-specific trends were identical. A significant p value (p < 0.05) indicates that the trends were not identical (ie, they had different incidence rates and coincidence was rejected).

d. Tests whether sex-specific trends were equal. A significant p value (p < 0.05) indicates that the trends were not equal (ie, they had different incidence rates and parallelism was rejected).

e. Multiple testing correction was applied with P-value cutoffs at 0.05 for all ages and the prespecified older and younger subgroups, and at 0.025 for the post-hoc 35-54 and 15-34 age subgroups.

Supplemental Table S2: Sex-specific Trends for restricted ICD-O-3 codes-based Pancreatic Ductal Adenocarcinoma (PDAC) and Pancreatic Neuroendocrine Tumor (PanNET) Stratified by Age Groups

		Trends					Comparison between sex-specific trends (p-value)[e]			
Age group (years)	**Number of cases (%)**	**Time period**	**APC[a] (95% CI)**	**APC p-value**	**AAPC[a] (95% CI)**	**AAPC p-value**	**AAPC difference[b] (95% CI)**	**AAPC difference**	**Test of coincidence[c]**	**Test of parallelism[d]**
Pancreatic Ductal Adenocarcinoma (PDAC)										
All Ages	**N = 665,913**									
Men	335,313 (50.4%)	2001-2007	1.22* (0.79-1.65)	<0.001	0.75* (0.59-0.91)	<0.001	0.06 (-0.17-0.28)	0.64	<0.001	0.20
		2007-2018	0.49* (0.35-0.64)	<0.001						
Women	330,600 (49.6%)	2001-2008	1.31* (0.97-1.65)	<0.001	0.69* (0.54-0.85)	<0.001				
		2008-2018	0.27* (0.09-0.44)	0.006						
Age ≥55	**N = 598,665**									
Men	295,575 (49.4%)	2001-2007	1.26* (0.80-1.72)	<0.001	0.83* (0.66-1.00)	<0.001	0.11 (-0.17-0.39)	0.43	<0.001	0.005
		2007-2018	0.60* (0.44-0.75)	<0.001						
Women	303,090 (50.6%)	2001-2006	1.60* (0.89-2.32)	<0.001	0.72* (0.50-0.94)	<0.001				
		2006-2018	0.35* (0.19-0.52)	<0.001						
Age 15-54	**N = 67,230**									
Men	39,731 (59.1%)	2001-2018	0.04 (-0.21-0.29)	0.75	0.04 (-0.21-0.29)	0.75	-0.83* (-1.21- -0.44)	<0.001	<0.001	0.002
Women	27,499 (40.9%)	2001-2018	0.86* (0.53-1.20)	<0.001	0.86* (0.53-1.20)	<0.001				
Pancreatic Neuroendocrine Tumor (PanNET)										
All ages	**N = 20,488**									
Men	10,974 (53.6%)	2001-2011	1.08 (-0.82-3.01)	0.24	11.79* (8.71-14.96)	<0.001	0.30 (-3.91-4.51)	0.89	<0.001	0.66
		2011-2014	60.53* (36.30-89.06)	<0.001						
		2014-2018	9.62* (6.35-12.99)	<0.001						

Women	9,514 (46.4%)	2001-2011	0.88 (-0.83-2.62)	0.28	11.48* (8.70-14.34)	<0.001				
		2011-2014	60.10* (38.04-85.68)	<0.001						
		2014-2018	9.11* (6.16-12.14)	<0.001						
Age ≥55	**N= 14,226**									
Men	7,953 (55.9%)	2001-2004	-7.97* (-15.90-0.72)	0.07	11.25* (8.68-13.88)	<0.001	-0.86 (-6.02-4.29)	0.74	<0.001	0.21
		2004-2011	3.95* (0.98-7.00)	0.02						
		2011-2014	56.95* (40.56-75.24)	<0.001						
		2014-2018	11.57* (9.39-13.79)	<0.001						
Women	6,273 (44.1%)	2001-2011	1.69 (-1.14-4.61)	0.21	12.11* (7.75-16.65)	<0.001				
		2011-2014	57.28* (24.78-98.25)	0.001						
		2014-2018	10.99* (6.54-15.63)	<0.001						
Age 15-54	**N= 6,231**									
Men	3,007 (48.3%)	2001-2011	0.61 (-2.72-4.06)	0.69	10.70* (5.01-16.70)	<0.001	0.14 (-6.88-7.16)	0.97	0.78	0.93
		2011-2014	60.91* (18.0-119.43)	0.007						
		2014-2018	6.17 (-0.22-12.97)	0.06						
Women	3,224 (51.7%)	2001-2011	-0.26 (-2.48-2.01)	0.80	10.56* (6.73-14.52)	<0.001				
		2011-2014	64.10* (33.40-101.87)	<0.001						
		2014-2018	6.37* (2.13-10.77)	0.007						

a. APC and AAPC refer to "annual percentage change" and "average annual percentage change," respectively.

* indicates a significant difference (p < 0.05). Trends were calculated using version 4.9 of the Joinpoint Regression program (National Cancer Institute). Up to four joinpoints (5 line segments) were allowed. The annual percentage change over the whole period (2001-2018) is equal to the average over all the subgroups.

b. Negative value indicates greater average APC in women.

c. Tests whether sex-specific trends were identical. A significant p value (p < 0.05) indicates that the trends were not identical (ie, they had different incidence rates and coincidence was rejected).

d. Tests whether sex-specific trends were equal. A significant p value (p < 0.05) indicates that the trends were not equal (ie, they had different incidence rates and parallelism was rejected).

e. Multiple testing correction was applied with P-value cutoffs at 0.05 for all ages and the prespecified older and younger subgroups.

Supplemental table S3: Sex-specific Trends for Young adult (aged 15 - 54 years) Biopsy Proven Pancreatic Ductal Adenocarcinoma (PDAC) Stratified by Histologic Groups

		**Trends**					**Comparison between sex-specific trends (p-value)[e]**			
**Histologic group**	**Number of cases (%)**	**Time period**	**APC (95% CI)[a]**	**APC p-value**	**AAPC (95% CI)**	**AAPC p-value**	**AAPC difference (95% CI)[b]**	**AAPC diff p-value**	**Test of coincidence[c]**	**Test of parallelism[d]**
**PDAC, NOS**	**N = 49,281**									
Men	28,939 (58.7%)	2001-2018	0.36* (0.05-0.68)	0.03	0.37* (0.05-0.68)	0.03	-0.66* (-1.09- -0.23)	0.003	<0.001	0.15
Women	20,342 (41.3%)	2001-2018	1.02* (0.68-1.37)	<0.001	1.02* (0.68-1.37)	<0.001				
**Mucinous**	**N= 2,642**									
Men	1,297 (49.1%)	2001-2018	-6.23* (-7.16- -5.29)	<0.001	-6.23* (-7.16- -5.29)	<0.001	-0.47 (-2.08-1.14)	0.57	0.62	0.53
Women	1,345 (50.9%)	2001-2018	-5.75* (-7.21- -4.28)	<0.001	-5.75* (-7.21- -4.28)	<0.001				

a. APC and AAPC refer to "annual percentage change" and "average annual percentage change," respectively.

* indicates a significant difference (p < 0.05). Trends were calculated using version 4.9 of the Joinpoint Regression program (National Cancer Institute). Up to four joinpoints (5 line segments) were allowed. The annual percentage change over the whole period (2001-2018) is equal to the average over all the subgroups.

b. Negative value indicates greater average APC in women.

c. Tests whether sex-specific trends were identical. A significant p value ($p < 0.05$) indicates that the trends were not identical (ie, they had different incidence rates and coincidence was rejected).

d. Tests whether sex-specific trends were equal. A significant p value ($p < 0.05$) indicates that the trends were not equal (ie, they had different incidence rates and parallelism was rejected).

e. Multiple testing correction was applied with P-value cutoffs at 0.05 for all ages and the prespecified older and younger subgroups.
